# Conflict affected, parallel health systems: challenges to collaboration between ethnic and government health systems in Kayin State, Myanmar

**DOI:** 10.1186/s13031-021-00396-z

**Published:** 2021-07-28

**Authors:** Colette Pang Biesty, Aung Ja Brang, Barry Munslow

**Affiliations:** The University of Sheffield Medical School, Sheffield, UK

**Keywords:** Myanmar, Kayin/Karen state, Devolved healthcare, Access to healthcare, Universal health coverage, Health as a bridge to peace, Ethnic health system, Indigenous health, Health system strengthening

## Abstract

**Background:**

Myanmar has had a long history of civil wars with its minority ethnic groups and is yet to see a sustainable peace accord. The conflicts have had a significant impact on health in Myanmar, with ethnic populations experiencing inequitable health outcomes. Consequently, to meet the health needs of ethnic people, Ethnic Health Organisations and Community-Based Health Organisations (EHO/CBHOs) created their own health system. The EHO/CBHO and Government health systems, provided by the Myanmar Ministry of Health and Sports (MoHS), remain parallel, despite both stakeholders discussing unification of the health systems within the context of ongoing but unresolved peace processes. EHO/CBHOs discuss the ‘convergence’ of health systems, whilst the MoHS discuss the integration of health providers under their National Health Plan.

**Methods:**

A qualitative study design was used to explore the challenges to collaboration between EHO/CBHOs and the MoHS in Kayin state, Myanmar. Twelve health workers from different levels of the Karen EHO/CBHO health system were interviewed. Semi-structured, in-depth interviews were digitally recorded, transcribed, and coded. Data was analysed thematically using the Framework method. Topic guides evolved in an iterative process, as themes emerged inductively from the transcripts. A literature review and observation methods were also utilised to increase validity of the data.

**Results:**

The challenges to collaboration were identified in the following five themes: (1) the current situation is not ‘post conflict’ (2) a lack of trust (3) centralised nature of the MoHS (4) lack of EHO/CBHO health worker accreditation (5) the NHP is not implemented in some ethnic areas.

**Conclusions:**

Ultimately, all five challenges to collaboration stem from the lack of peace in Myanmar. The health systems cannot be ‘converged or ‘integrated’ until there is a peace accord which is acceptable to all actors. EHO/CBHOs want a federal political system, where the health system is devolved, equitable and accessible to all ethnic people. External donors should understand this context and remain neutral by supporting all health actors in a conflict sensitive manner.

## Background

### Political background

Myanmar has a long history of civil war between ethnic armed organisations (EAOs) and the Tatmadaw, the armed forces of Myanmar. Civil war with the Karen National Union (KNU) non-state armed group in Kayin state is one of the oldest insurgencies in Myanmar [1]. This has had a severe impact on the provision of healthcare and other services in these politically contested territories [2] as 50 years of military dictatorship has left the provision of health services a low priority [3].

With the transition to a quasi-civilian government in 2011 under Thein Sein, state level ceasefires were signed with various EAOs, including the KNU on January 12th, 2012 [1]. More recently the Nationwide Ceasefire Agreement (NCA) was signed in 2015 between eight armed organisations, including the KNU, and the Government [4]. Continued conflict between EAO NCA signatories (EAO-NCA) and the Tatmadaw has led to absent or inaccessible Government health-services for some ethnicities, severely setting these regions back in reaching World Health Organisation’s (WHO) health outcome goals [5].

The 2015 NCA appears unsustainable with continued fighting accompanying peace-talks between signatories, non-signatories and the Tatmadaw [4]. During the twenty-first Century Panglong Conference that endeavoured to bring peace in July 2018, clashes between the Tatmadaw and the KNU occurred following a growing government military presence in restricted KNU controlled areas [6]. On October 27th 2018, the KNU withdrew from the formal peace process as talks had stagnated [7]. Within this political context the current study endeavours to explore the complex ecosystem of health actors in Kayin state, Myanmar.

Fighting and displacement continue in Kayin State throughout the COVID-19 pandemic of 2020 [8]. Despite KNU and other EAOs calling for a nationwide ceasefire to collectively fight coronavirus [9], there have been reported cases of the Tatmadaw burning down KNU’s Covid-19 Screening Posts [10]. However, data collection and analysis for this study was conducted before the COVID-19 crisis.

### Impact on health services

Civil war has a significant impact on health systems. In 2009, Myanmar’s health sector expenditure was the lowest in the world: 0.2% of the Gross Domestic Product [11]; 50% of children were stunted; and life expectancy was 56 years of age [12]. Health expenditure has increased a little since then [5] with an increase in health work force numbers [13]. However, the provision of health services in Myanmar remains inequitable between: the urban and the rural; the rich and the poor; disadvantaged groups; and populations in conflict-affected areas [14].

Multiple health actors provide healthcare in Myanmar, such as: the Government’s Ministry of Health and Sports (MoHS); private-for-profit providers; National Non-Governmental Organisations (NGOs); International Non-Governmental Organisations (INGOs); Ethnic Health Organisations (EHOs) and Community-Based Health Organisations (CBHOs) [11, 13]. Out-of-pocket payments remain high when accessing health services in the MoHS and the private sector [14].

EHOs were established by EAOs to operate as a health department in order to make healthcare more accessible to their vulnerable populations. The KNU’s EHO, the Kawthoolei/Karen Department of Health and Welfare (KDHW), works to provide healthcare to people governed by the KNU. Alongside EHOs, CBHOs also developed and work in partnership with EHOs but remain independent from EAO governance structures. CBHO service users are in hard-to-reach and often ethnic areas and are not exclusive to any specific ethnic group or EAO governance territories.

EHO/CBHO services are provided free of charge as many ethnic people in Kayin State find Government health services inaccessible [5]. This can leave EHO/CBHOs dependent on donors to fund their work. These health services remain as a parallel health system to that of the Government’s MoHS [2].

### Working together

Both the Government’s MoHS and EHO/CBHOs have publicly expressed the benefits of working together to provide comprehensive healthcare for all in Myanmar. The MoHS launched the National Health Pan (NHP) in December 2016, to try and combat the issues of: weak health infrastructure; lack of human resources; high out-of-pocket spending; and inequitable distribution of health services [11]. The NHP proposed to merge all health actors with the MoHS health system in a purchaser and provider relationship. This is where the MoHS would strategically ‘purchase’ the services of EHO/CBHOs, NGOs and private health providers, to improve access to a basic Essential Package of Health Services (EPHS), unrealistically intended to be achieved by 2020. Funding to purchase health providers outside of the MoHS is to come from donors and development partners, until it can be transferred back to the MoHS and be managed by inclusive autonomous boards [11]. The NHP hopes to achieve Universal Health Coverage (UHC) in Myanmar by 2030, with UHC ‘defined as all people having access to needed health services of quality without experiencing financial hardship’ [11].

An alternative vision is provided by EHO/CBHOs, who have united to form the Health Convergence Core Group (HCCG). They have put forth the agenda of health system ‘convergence’, the ‘systematic, long term alignment of Government, ethnic, and community-based health services’ [15]. EHO/CBHOs want to align Government and ethnic health systems in a manner that clearly takes into account the current political process. The convergence of health systems we will argue, is very much dependent on the political process between the Government and EAOs. This has been depicted by the ‘rocket ship model’ attached in the Appendix as Appendix.

Although both healthcare stakeholders have recognised the need to collaborate, there is disagreement regarding the methodology of unification and collaboration between health systems. The NHP intends to integrate the EHO/CBHO network, whilst the HCCG proposes a power sharing health system where more policies are made at state rather than national level [16]. Both health systems have strengths and weaknesses. The MoHS has access to more resources, whilst EHO/CBHOs has more access to rural service users in ethnic regions of the country, as well as an understanding of their health needs [2]. Therefore, collaboration and convergence of health systems could significantly improve health outcomes and health equity throughout the country. Given the mutual recognition of the benefits of collaboration, what is needed is consensus as to the methodology of working together. This research hopes to explore the experiences of health workers in the ethnic health system and elucidate what they feel are the challenges to collaboration between ethnic and Government health systems.

## Methods

### Research aim and objective

The research aim was to explore challenges to collaboration between ethnic health workers and the Government health system in Kayin state Myanmar. The primary objective of the study was to explore perceptions of ethnic Kayin health workers of the challenges of integrating their Ethnic and Community Based Health Organizations effectively with the Government health system.

### Study design and setting

A qualitative study was chosen to explore the challenges to collaboration that EHO/CBHO health workers experience as it allows for a naturalistic exploration of a participants personal beliefs [17]; and is recommended when exploring the organisation of health services in times of political reform [18]. Semi-structured, In-depth interviews with 12 key informants in the Ethnic Health System were conducted. All interviews were conducted in Mae Sot, Thailand between the 18th June 2019 and the 1st of August 2019. This study was hosted by Back Pack Health Worker Team (BPHWT), a CBHO who have been providing healthcare to conflict-affected and rural areas in Myanmar since 1998. With headquarters in Mae Sot, BPHWT are a multi-ethnic organisation operating throughout Myanmar, covering a population of 298,273 people as of 2018 [16]. BPHWT were vital to finding participants for this study and helping the researcher contextualise the findings. This included attending seminars, talks and events organised for BPHWT staff as well as providing valued feedback throughout the research project.

### Sampling

Participants were purposively selected, supported by a senior BPHWT staff member who was informed of the sampling frame and helped to introduce the researcher to leaders in the EHO/CBHO health system. A snowball sampling method was also used to access field health workers who are temporarily visiting the Mae Sot central office for training or data collection [19]. A total of 12 key informants were interviewed from different managerial positions within the Ethnic Health System. This research also endeavoured to get a gendered balance of perspectives, however this was dependent on who held these positions and health worker availability. Table 1 provides more information:

**Table 1:** Participant Information

Position	Organisation	Number of Participants	Gender Balance
Senior Leadership	Back Pack Health Worker Team	2	2 M
	Burma Medical Association	1	1 M
	Ethnic Health System Strengthening Group	1	1 M
	Karen Department of Health and Welfare	1	1 M
Central Office Coordinators	Back Pack Health Worker Team	3	3F
Field Health Workers	Back Pack Health Worker Team	4	1F 3M
**Total**		**12**	**4F** **8M**

The original study design included MoHS participants, however security concerns for participants and the research team were flagged at the Liverpool School of Tropical Medicine Masters Review Panel. Therefore, interviews with MoHS health workers were unfortunately out of the scope of this study. The lack of the MoHS perspective is a real limitation and clarified further in the discussion.

### Data collection

Semi-structured in-depth interviews were conducted by the first author (CB) in English with participants who chose to speak in English. Most interviews were conducted in Burmese with the first author (CB) and the second author (BAJ) as an interpreter. Both authors discussed the aims of the research and qualitative methodology prior to starting interviews. Interviews were conducted collaboratively between CB and BAJ. The topic guides were developed by (CB), then iteratively and collaboratively evolved to ensure key topics were covered and relevant. Topic guides included (1) the current ethnic health system (2) views of the MoHS health system (3) experiences working with the MoHS (4) universal health coverage through convergence of health systems. The topic guides were initially translated by an independent Burmese translator, and then back translated by the second author to ensure questions were framed appropriately.

All interviews were recorded, and the English translation was transcribed verbatim by the first author (CB) to aid with data immersion. Throughout the research process, (CB) kept a reflective journal to assess positionality and would regularly meet with (BAJ) and BPHWT staff to reflect on emerging themes.

### Data analysis

An iterative thematic analysis using the Framework Method was used to analyse the data as outlined by Gale et al. [20]. Through a triangulation of data sources: from interviews with key informants in different positions of the ethnic health system, to analysing BPHWT annual reports and MoHS official documents, the researcher was able to check the validity of the findings [17].

Open codes were applied to the first 5 transcripts, both inductive and topic guide based deductive codes were used. A working analytical framework was then developed on excel and manually applied to the transcripts. After discussion with authors and BPHWT staff, the analytical framework was then revised, and a final framework developed. Participants based in Mae Sot and who have access to emails were able to validate findings, however participant checking with field health workers was difficult due to lack of email access and unavailability whilst conducting duties in the field.

### Researcher positionality

Awareness of the researcher’s positionality and their impact on data collection and analysis [17] meant a reflexive approach to the study was preferable. At the time of research, CB and BM were independent and external researchers, whereas BAJ was a former staff member of BPHWT. Mitigation of researcher bias was attempted through variability of ‘insider’ and ‘outsider’ perspectives, alongside consistent self-assessment and discussion of positionality and assumptions to enhance validity of the results.

### Ethics

This research project was approved by the Liverpool School of Tropical Medicine Masters Review Panel in April 2019 (Ethics Application 1926), and the Mae Sot Community Ethics Advisory Board in June 2019 (Ethics Application 1904). All participants received a participant information sheet before the interview. All respondents have provided informed written consent to participating in the study. The voluntary nature of the study and the participant’s rights to their data was also reiterated at the beginning of each interview.

## Results

Participant’s experiences and perceptions regarding the challenges to collaboration between ethnic health workers and the Government health system can be divided into the following five main themes. Table 2 presents the themes and sub-themes of the study.

**Table 2:** Themes and Sub-themes of the Study

Themes	Sub-themes
1. Non ‘post-conflict’ context	a. Unstable Ceasefire
	b. No Political agreement reached
	c. International Actors and their impact
2. Lack of trust	a. Between ethnic communities and the Government
	b. Between conservative district administrators and military brigades of the KNU and the Government
	c. Between EHO/CBHO health workers and the MoHS
3. Centralised nature of the MoHS	a. Challenges in human resource management
	b. Challenges in centralised decision making
	c. Need for a decentralised federal health system
4. Lack of EHO/CBHO health worker accreditation	a. Challenges in referral
	b. Challenges in immunisation
	c. Lack of access to MoHS recognised training
5. NHP is not implemented in some ethnic areas	a. Difficult to implement NHP in a conflict context
	b. EHO/CBHOs strongly disagree with their ‘provider’ position

### Non ‘post-conflict’ context

A key theme that emerged is that the current situation in Myanmar cannot be considered as a ‘post-conflict’ situation. Informants stressed that there is an unstable ceasefire and no political agreement. Yet international actors have engaged as if it were a post-conflict situation. These factors combined has contributed to the difficulty in bringing the two health systems together.

### Unstable ceasefire

Respondents clearly say that the NCA has not ceased conflict in Kayin State. As one EHO/CBHO leadership member states:

‘*Even if there is a National Ceasefire Agreement… there is still conflict. More conflict or less conflict, there is no post-conflict period.’ (IDI4, Leadership).*

This significantly limits the extent of collaboration that can occur between EHO/CBHOs and the MoHS. There continues to be violations to the Nationwide Ceasefire Agreement, with displacement and outbreaks of fighting, especially in northern Kayin state [8, 21, 22]. EHO/CBHO health worker safety remains under threat:

*‘The ceasefire (is) not too strong right now, nearly broken. We don’t know, but maybe they will blame our health workers as insurgents … not only [in] Karen state, but also Palaung (Northern Shan State) or Kachin state where they are arrested, Rakhine state as well.’ (IDI3, Leadership).*

Before the ceasefire in Kayin state, Government authorities had perceived EHO/CBHO health workers as EAO insurgents. BPHWT health workers had been detained under the Unlawful Associations Act. EHO/CBHOs were removed from the list of unlawful associations when they signed the NCA in 2015 [2]. But the unstable ceasefire once again places health workers at risk of detention, and MoHS staff reluctant to collaborate. Moreover, the Covid-19 crisis has shown that the unstable ceasefire has directly affected the ethnic health system with the destruction of the KNU’s Covid-19 screening posts [23].

### No political agreement reached

Interviewees argued a ceasefire does not equate to peace. Political dialogue between the Government and the KNU remains deadlocked. Consequently, Kayin state cannot be considered as a post-conflict situation, hence the convergence of health systems should not be encouraged until there is a political peace accord:

*‘The ceasefire is not peace. The ceasefire just stops the fighting... international understanding of a post-conflict situation is totally wrong.’ (IDI5, Leadership).*

Without a sustainable peace accord, working with governmental bodies remains difficult. Some in the leadership of EHO/CBHOs believe the lack of a political agreement reflects a disconnect between the Tatmadaw and the National League for Democracy (NLD) Government:

*‘There are two governments, civilian and military ... Almost every important ministry is controlled by the military … The civilian Government have a statement on the peace process … at the same time the military also have a position on the peace process.’ (IDI4, Leadership).*

The civilian Government have no jurisdiction over the Tatmadaw under the 2008 constitution and there continues to be opposition between the Tatmadaw and the NLD. Article 436 of the 2008 constitution states that any constitutional reform needs more than 75% of votes from Parliament and 25% of seats belong to the Tatmadaw. Without the will of the military to find a political agreement with EAOs, it is unlikely that the political process will progress, as one respondent elucidates:

*‘Ethnic groups require [that] they have rights, they have opportunities, they wanted democracy and peace … But the Government Army’s interests are not the same. … Peace, Democracy, freedom in Myanmar is so difficult.’ (IDI7, Central Office Coordinator).*

Lack of political agreement between the KNU, the Government, and the military hinders the two health systems converging. The health sector of the KNU, the KDHW, and their affiliated CBHOs cannot move ahead of the peace process and integrate with the MoHS as laid out in the NHP:

*‘We don’t want to be a parallel system, but at the moment [the EHO/CBHO health system] stays as a parallel system. However maybe one day, if our country becomes fully [peaceful], maybe then the system will come together as convergence, the best system for all. Not the existing system. Not as a MoHS system. Not centralised, only decentralised. The best system that works for our country, this should be aligned with the federal system, this is our aim.’ (IDI5, Leadership).*

For respondents, the ethnic people of Myanmar want a federal political system where all ethnicities are represented and have more autonomy over how they are governed. Ethnic health workers want a health system where health system governance is also devolved. EHO/CBHOs have better access to ethnic populations and understand their health needs. Unless the principles of federalism are met, EHO/CBHOs do not want to collaborate and become a ‘provider’ for services that the MoHS will ‘purchase’ as stated in the NHP.

Separate geographical health areas will determine where EHO/CBHOs and the MoHS work. The KDHW, will continue to work in the KNU governed territories. CBHOs, like BPHWT, also often choose to work in these territories as they believe these territories have the most unmet health needs:

*‘Some parts of (BPHWT) have to work together with the Ethnic Armed group control area, because the Government side have nothing to provide for that (area). You might hear the 4 cuts policy … cut off food, cut off money, cut off recruitments, cut off information. That is why the community [do not] receive any of what they need … That is why... we provide.’ (IDI1, Leadership).*

Without a political agreement, parallel governance structures will also be maintained, with EHO/CBHOs and the MoHS targeting different geographical catchment areas. These spatial divisions will continue to be a challenge to collaboration between the ethnic health workers and the MoHS.

### International actors and their impact

Informants stressed that it is crucial for international agencies and donors to understand that without a genuine peace accord Kayin State is not a post-conflict situation. Although a civilian Government was elected in 2015, it is one of many actors involved in the ongoing Myanmar peace process. Some international actors portray the Government as the sole decision maker, or purchaser, of health services for the country:

*‘International Government donors are trying to support strengthening the system and the development of Myanmar because of the landslide election of the NLD. So, every agency and international NGOs … said that they would be supporting the NLD National Health Plan.’ (IDI11, Leadership).*

This diversion of funding to support the Government’s MoHS has significantly reduced the funding to EHO/CBHOs:

*‘Our program planning (is) not enough for requirements (with) … budget limitations and donors interrupting their projects.’ (IDI7, Central Office Coordinator).*

Donor support for the NHP means the EHO/CBHOs must access funds through the Myanmar Government. EHO/CBHO leadership have stated that this is a challenge and leaves EHO/CBHOs reliant on external donor funds. The shift of donor funds towards the NHP is seen as pressure to integrate with the MoHS health system before a peace accord is reached. One participant argues that the Myanmar Government should not be supported until there is improvement in human rights, progression of the peace process, and democratisation of Myanmar:

*‘International governments should [monitor these] three main indicators: human rights violations; peace process; and democratisation. So, if three things have not improved, they shouldn’t directly support the Burmese Government.’ (IDI3, Leadership).*

EHO/CBHOs worry that the way in which some international actors have engaged with health systems strengthening in Myanmar has been one-sided, having wider political repercussions in an ongoing conflict. Genuine integration of health systems will remain dependent on the achievement of a sustainable political peace agreement.

### Lack of trust

The research revealed a lack of trust in the Government administration among ethnic people, between: ethnic communities and Government officials; EHO/CBHO health workers and the MoHS; as well as between some district administrators and military brigades of the KNU and the Government. This represents a significant challenge to collaboration between ethnic health workers and the Government health system.

### Between ethnic communities and the government

The civil war and human rights violations against the non-Bamar ethnicities has created a lack of trust in the Government among the ethnic communities that EHO/CBHOs serve. One participant details their experience in the field:

*‘As soon as they hear a Burmese name, or that the Burmese are coming, they will just think of the old times when the military comes and destroys the village. There are still many cases when (*EHO/CBHO *staff) told the patients to go to the (MoHS) hospital [but] the patient still ignores going to the hospital, because they do not trust the Government yet.’ (IDI9, Field Health Worker).*

There has been a prolonged historical separation between ethnic and Government governing structures. Ethnic community trust in the Government, and therefore the MoHS, is essential in order for MoHS staff to work alongside EHO/CBHOs in ethnic communities. Without peace and reconciliation, ethnic communities remain apprehensive about any form of Government administration, including those of the health sector.

### Between Conservative District administrators and military brigades of the KNU and the government

KNU has a decentralised administration structure, therefore different areas are governed by different KNU district administrators and military brigades who have decision making power over their controlled areas. Some conservative KNU district administrators and military brigades deny access to any Governmental bodies who wish to enter their territories. The increased presence of the MoHS was perceived to be a form of Government administration expansion. Regions under complete KNU control rely on EHO/CBHOs to meet the ethnic people’s health needs. One respondent working as a health worker in a conservative region states they have not worked with the MoHS staff due to KNU policies:

*‘Currently they do not have any staff coming from the Ministry of Health and Sports... The reason KNU have that policy is because they are concerned about the expansion of Government administration to their controlled areas.’ (IDI2, Field Health Worker).*

In territories of mixed EAO and Government control, some KDHW branches have a different stance and work more closely with the MoHS on some programs:

*‘Mixed area means you have the Government administration, you have the KNU administration. So, there’s no way to stay away from each other … in mixed areas we have more collaboration between MoHS and the KDHW health workers.’ (IDI11, Leadership).*

Despite this pragmatic approach to collaboration with the Government, there continues to be breaches in trust. This is due to the violations of the ceasefire such as the building of MoHS ‘Rural Health Centres’ (RHCs) in ethnic areas, one respondent elaborates:

*‘Government do not respect the ethnic territories. They come and build the Rural Health Centre, or the Sub Health Centre in the ethnic areas, they do not respect, do not inform, do not consult with us … we feel like they do not respect our administration.’ (IDI11, Leadership).*

In the current climate of Covid-19, the Tatmadaw have stated KNU’s screening posts breach the NCA [23], whilst the KNU have stated the Tatmadaw have violated the NCA by forcing ethnic health workers to leave checkpoints, and burning down some screening posts [24]. Action by the Tatmadaw and the Government that violates the NCA will continue to engender a lack of trust among some brigades of the KNU and the Government.

### Between EHO/CBHO health workers and the MoHS

Some EHO/CBHO health workers also felt apprehensive about the MoHS and their intentions, especially with the building of RHCs. One field health worker explains that the centres are not always well resourced:

*‘The Government staff they’re trying their best, but … in some areas they have very good infrastructure … But inside the building there’s no medical supplies or medical staff.’ (IDI2, Field Health Worker).*

Two respondents share their confusion and concern over the Government’s intentions:

*‘There are 9 health facilities that they built. Just close to the existing [ethnic] health organisation’s health facilities. So, does that mean they built it for their own people, or do they come to take the area?’ (IDI4, Leadership).*

*‘Government come and build an RHC, but they did not assign any people, no medicine, no staff. We are also wondering whether we should take this facility back.’ (IDI11, Leadership).*

Some ethnic health workers have said that MoHS staff visit the RHCs periodically to provide outreach services, as this health worker explains:

*‘They come to the [Rural] Health Centre only once a month. And once a month they will stay in the centre for 2 or 3 days. And when they come, they just take the data from them. How many ANC (antenatal care patients) ...Then the next month they will come and give the ANC (antenatal care) care to that patient.’ (IDI9, Field Health Worker).*

This would be aligned with the MoHS strategy, as the NHP states that services can be provided on a scheduled basis through outreach services until a more permanent solution is found [11]. Depending on the region, some ethnic health workers have good relationships with MoHS staff who work at these RHCs, as this respondent describes:

*‘In some areas they have good relationships [with RHC staff] and sometimes they even work together. For example, giving health education in the school.’ (IDI9, Field Health Worker).*

Ethnic health workers and MoHS staff do not have a good relationship in other areas, given the expansive nature of the MoHS in taking over ethnic health facilities, as explained by the respondent:

*‘In some cases, before the ceasefire, Back-Pack team was already in the village and they worked together with the village community, and then they set up a clinic … But after the ceasefire, Government administration came in and take over the building, and then they named it RHC (Rural Health Centre) … After that, they stopped working together.’ (IDI9, Field Health Worker).*

RHCs are one example of how a lack of trust between ethnic health workers and MoHS health workers can be propagated if strong relationships aren’t built. The MoHS’ actions can sometimes be perceived as expansive and controlling. One participant notes the negative ‘competing spirit’ between the MoHS and EHO/CBHOs during a humanitarian response to flooding in Kayin state.

*‘At that time Back-Pack tried to support [with items] like rice, basket, some donations... As soon as Back-Pack arrived … the Burmese Government also came and gave donations, but with very little support like three packs of [instant noodles] … the competing spirit is still going on.’ (IDI8, Central Office Coordinator).*

Many participants agreed that trust building between health workers in the EHO/CBHOs and the MoHS is crucial. This will take time as the political and health systems have been apart for many years as these two respondents explain:

*‘I think it will take time to know each other, [there’s] a need to build trust … .being apart for years … So, it’s not easy to trust each other.’ (IDI4, Leadership).*

*‘It’s been many years that we are under different systems … So, trust building is the main obstacle for collaboration.’ (IDI6, Central Office Coordinator).*

Building trust and communication between health workers is key to bringing people together after working in different systems for years. If Myanmar reaches a sustainable peace agreement, peace and reconciliation can begin.

### Centralised nature of the MoHS

Another challenge to collaboration is the highly centralised structures of the MoHS. Participants stated that the 2008 constitution keeps decision-making and resource sharing at the Nay Pyi Taw central level. It leaves the MoHS at State and Township levels with insufficient resources and disempowered to make their own decisions, such as the decision to collaborate with local EHO/CBHOs. Participants felt the challenges to collaboration also stem from the centralised MoHS health system and will persist until there is constitutional change that allows a devolved health system.

### Challenges in human resource management

MoHS manages its human resources in a centralised manner posing challenges in local language communication and staff turnover. The MoHS staff pool are licensed and appointed at the central level in Nay Pyi Taw:

*‘(Concerning) recruitment for Government medical doctors, the state level cannot decide. That has to be appointed by the central level at Nay Pyi Taw … auxiliary midwife and also nurses must be appointed by the central level... also the key positions, the central level keep.’ (IDI1, Leadership).*

Participants explained how this approach hinders the recruitment of an inclusive and diverse health workforce who can communicate with ethnic health workers, and ethnic populations. Participants from all levels of the EHO/CBHO health system describe how some MoHS health staff, working in ethnic areas, do not understand the local languages. A centralised pool of staff is distributed throughout the country, sometimes leading to language barriers with local populations. Some ethnic health workers may only speak Karen and not Burmese, leading to communication problems between health workers:

*‘But in the ethnic side also, language is a problem... they cannot speak the Burmese language … most of the Government health staff speak Burmese … they also bring staff from the other areas to the Karen state ethnic areas. So that’s why the communication is also very [difficult].’ (IDI5, Leadership).*

This hinders patient communication and limits building relationships between ethnic health workers in the EHO/CBHO health system, and health workers in the MoHS. Hospitals who have local language capacity have an improved relationship with ethnic communities, from both ethnic patients and ethnic health workers:

*‘It also depends on the hospital. The [X: name redacted] hospital is getting better because … if the patients cannot speak Burmese, they will talk with them in Karen. But for the hospital in [Y: name redacted], they do not have such things. And the relationship with them is still quite bad.’ (IDI9, Field Health Worker).*

The centralised distribution of non-local staff can also lead to a high turnover of MoHS staff in ethnic communities due to communication issues or being reassigned to different areas of the country. Participants have experienced MoHS staff who have been committed to working with the EHO/CBHO health system, however high turnover of MoHS staff makes building relationships difficult as these respondents describe:

*‘Once (there is) collaboration, they are removed from that post to go somewhere else... Within 10 years here, I’ve been in contact with 4 Karen State Health and Support directors... Sometimes (for) only 1 year, then they move from here to somewhere.’ (IDI4, leadership).*

*‘If the doctor is good in making relationships, then their staff and all other things, referrals … they are all okay. Sometimes when the doctor has built good relationships with [*EHO/CBHOs*], then suddenly the doctor has been moved to another place.’ (IDI6 Central Office Coordinator).*

Establishing relationships and trust are crucial for collaboration. Unfortunately, once a relationship is established, MoHS staff may be reassigned due to the centralised nature of human resource management in the MoHS.

### Challenges in centralised decision making

Decision-making and governance also follows a centralised and top-down approach that deters local collaboration:

*‘Sometimes the State level and Township level authorities want to try (to collaborate), but just because of the system they don’t dare to do it … the system blocked their ability to do collaboration.’ (IDI4, Leadership).*

*‘The health workers from the Government side would like to help the people as well, they have a heart. So, they also want to (collaborate), but they do not have decision to do (so).’ (IDI5, Leadership).*

All health workers share the same goals, to improve the health outcomes of the people they serve. But the centralised system stops relationships between MoHS and EHO/CBHO health workers being built and has caused delays in implementing collaborative programs as this respondent explains:

*‘They always say... “we have to wait till the command, from the top level” … it creates the delay [in] implementation of any project.’ (IDI11, Leadership).*

### Need for a decentralised, Federal Health System

EHO/CBHO leaders argue that a decentralised health system is key to improving the Myanmar health system. Decentralisation in the management of human resources would allow a more diverse health workforce that could communicate with targeted populations. Management of the health workforce at state level could also improve the staff vacancies as this respondent explains:

*‘State level must have the state Health Minister. If not, everything has to be … from above … they need to work together … on how to solve any vacant places, how to fill in the gaps.’ (IDI1, Leadership).*

EHO/CBHOs strongly believe in community involvement when planning health services for their targeted populations. This respondent envisions a health system that allows local empowerment as seen in a decentralised health system:

*‘Even if we have the peace or Federal Union, the community involvement is important. Local management power is important. If not, I think the funding is not sustainable. So that’s why [a] decentralised health system [would] share the power to the locals. The burden for the Government is maybe reduced.’ (IDI5, Leadership).*

Stated clearly by one respondent:

*‘To change the system, we have to change the constitution.’ (IDI4, Leadership).*

Until such change can occur, leadership of the EHO/CBHO health system do not want to integrate with a centralised health system. EHO/CBHOs are already practicing a decentralised, federal health system whereas the centralised nature of the MoHS goes against the principles of federalism as elaborated by the respondent:

*‘As multiple ethnic groups form networks to set up our own infrastructure, our own health system … we believe in a Federal Union, we try to build up our own system based on the federal principles … We have (already) started to practice the federal guiding principles.’ (IDI4, Leadership).*

Health workers in all levels of the ethnic health system have experienced difficulties working with a centralised health system. Centralisation goes against the EHO/CBHOs fundamental principles of local empowerment.

### Lack of EHO/CBHO health worker accreditation

Health workers in the EHO/CBHO health system continue to lack Government accreditation. This has caused difficulties in collaboration between the two health systems, especially in referrals of patients and immunisation programs.

### Challenges in referrals

When an EHO/CBHO health worker, from BPHWT, wants to refer a patient to a MoHS hospital, they write a letter that includes the history of the patient, examinations, and treatment that has been given. This is followed up by another letter from a Village Health Committee (VHC) member (Central Office Coordinator, IDI 6). When the letters are then given to MoHS staff, field health workers have had mixed experiences due to their lack of MoHS recognised accreditation as one health worker elaborates:

*‘ … [we] take the patients and send them to hospital and give the referral letter. Most of the time [MoHS staff] do not look at the referral letter as they do not recognise [us] as health workers … now, it’s a bit better, because some health staff start to look at the referral letter.’ (IDI10, Field Health Worker).*

Building professional and respectful relationships between health workers is key for collaboration and essential for the convergence of health systems. However, respondents often felt as if some MoHS staff ‘look down’ on EHO/CBHO health workers. Although EHO/CBHOs provide comprehensive training programs, participants state that they lack an MoHS recognised license or certificate to practice. This continues to deter grassroots collaboration between the two health systems:

*‘As a field worker [I] believe the coordination at the field level is not quite okay because the Government staff look down on [*EHO/CBHO *health workers]. Because they do not have any proper training, and they are not from [a University], they are not graduates, they only have hands on experience.’ (IDI10, Field Health Worker).*

Participants suggests MoHS staff only require more information about the Ethnic Health System and to meet with EHO/CBHO health workers more often to improve the relationship between health workers:

*‘Once we had a collaboration meeting...they got to know what we are capable of doing … The more you have collaboration meeting, I think the more understanding we will have.’ (IDI4, Leadership).*

As more communication and coordination between the two health systems occur, an understanding of each other’s knowledge and skill sets will help bridge the lack of accreditation until EHO/CBHOs have more access to MoHS recognised training. Such as, the joint training of auxiliary midwives by BPHWT, MoHS Karen State Department of Health, and Phlon Education Development Unit [25].

### Challenges in immunisation

Immunisations are essential for the ethnic community and requires working with the Government. Vaccinations are provided through the Government under its’ Expanded Program for Immunisations (EPI). UNICEF, a major contributor of vaccinations, has stated that the lack of infrastructure and coordination between the different health systems can be solved through strengthening the MoHS and supporting its’ EPI. Therefore in 2018, ‘GAVI, the Vaccine Alliance, signed a second phase agreement with MoHS, UNICEF and WHO to invest US$ 60 million in health system strengthening’ [26]. However, informants encountered challenges when working with the MoHS to immunise ethnic communities:

*‘The Government still do not recognise the health workers from Back-Pack team … in some areas, they do not contact the health staff from Back-Pack. They just come by themselves and they give the immunisation. The problem is that not all the children in that village receive the immunisation because the locals do not participate.’ (IDI6, Central Office Coordinator).*

Ethnic communities trust local ethnic health workers more and participate in MoHS led initiatives when EHO/CBHO health workers endorse them. One respondent describes successful collaboration through joint EHO/CBHO and MoHS health education sessions to engender community trust in the MoHS health staff:

*‘Before the villagers do not dare to receive the vaccination from the Government side. But after they collaborated in health education, the villagers started to feel okay to accept the vaccinations.’ (IDI9, Field Health Worker).*

The MoHS and the EHO/CBHOs are both vital in improving nationwide access to vaccinations. Immunisations exemplifies why EHO/CBHO health workers need to be accredited by the MoHS:

*‘To get the vaccine, plus to provide the service to the communities, we have to get the training, you know trained by the Ministry of Health and Sports.’ (IDI11, Leadership).*

Trusting EHO/CBHO health workers and their ability to carry out immunisations is a positive aspect of moving towards recognition of EHO/CBHOs. Official recognition and accreditation is dependent on the political situation:

*‘Everything is related to the political. So, if there is not [a] stable [political situation], we cannot go through to the negotiations, the recognition and accreditation process officially yet.’ (IDI11, Leadership).*

### Lack of access to MoHS recognised training

EHO/CBHO health workers need MoHS training:

*‘We don’t have qualifications like a medical university graduate...Because our people do not have a chance to go to the Government facilities... But if we are given a chance, just give us 10 years with some amount of money, we can do it.’ (IDI4, Leadership).*

There are no teaching hospitals in ethnic areas to improve the skills of their health workers. Instead, partnerships with Thai Universities and some Universities in Myanmar have been made to help improve the EHO/CBHO training curriculum so that it is equivalent to the MoHS. Moreover, one participant explains that attending Universities and training courses alongside MoHS staff could also help improve the relationship between health workers:

*‘Maybe [when] some of our staff have graduated, maybe they can work together with the MoHS staff inside... study together, maybe get closer and more familiar with each other, learning together. So, when they’re back, they can engage in the community level [and] in the Township level. So, this is what we hope for them.’ (IDI5, Leadership).*

Leadership have stated the importance of upskilling EHO/CBHO health workers in order to strengthen the Ethnic Health System and improve collaboration between EHO/CBHO health workers and MoHS health workers.

### NHP not implemented in some ethnic areas

The NHP supports engaging with EHO/CBHOs in order for Myanmar to reach UHC by 2030. However, some EHO/CBHO health workers are yet to experience engagement as envisioned in the NHP:

*‘The National Health Plan lays down four pillars... And every pillar... (involves) work with Ethnic Health Organisations. For example, in Human Resources for health, they talk about how to do the accreditation or recognition for the ethnic health workers. For the infrastructure, how to support the ethnic health facilities... But in reality, it’s almost 3 years already, I don’t see anything happen in the area where people work’ (IDI4, Leadership).*

EHO/CBHO field health workers from both KNU and mixed governance areas share their experiences regarding the NHP:

*‘About the National Health Plan, [I] once heard about it in a workshop. And in that workshop [I] also heard that they would have cooperation or coordination with the Government side, but lately [we] haven’t heard anything about the coordination or cooperation. So, [I] think that the coordination or cooperation cannot start, cannot be initiated.’ (IDI2, Field Health Worker).*

*‘According to [my] experience, the Government never support the EHOs to implement any activities in the area. And also, in the field level … the Government are aiming to get Universal Health Coverage without financial hardship, but the people are facing inaccessibility and when they go to their health centre or hospital, they still need to give money.’ (IDI12, Field Health Worker).*

Some participants questioned the sincerity of the NHP and felt the EHO/CBHO name was only included in the NHP in order to improve MoHS access to international donor funds:

*‘Sometimes I think the Government is just using our name … to get attention from the outside … the international donors. (Although it is) one small step that the Ethnic Health Organisation’s name is on the paper... in reality there is no change, there is no support, there is no progress... just the Government using our name.’ (IDI4, Leadership).*

Participants from leadership to field health workers are yet to see real implementation of the NHP as envisioned by the MoHS.

### Difficult to implement NHP in conflict context

UHC is not thought possible in the current conflict context:

*‘The Government introduced the National Health Plan to go along with Universal Health Coverage... Universal Health Coverage would improve a country like Burma …*. *how can you do that if there’s fighting, if there is no peace?’ (IDI4, Leadership).*

The NHP depicts the Government as the primary actor to achieve UHC and side-lines EAOS and EHO/CBHOs. The ‘National Health Plan’ would not reach the whole of the nation and leave ethnic communities out of the envisaged health system:

*‘The National Health Plan doesn’t mean Nationwide Health Plan … It is similar to the Nationwide Ceasefire... I always describe it as a partially Nationwide Ceasefire.’ (IDI4, Leadership).*

### EHO/CBHOs disagree with their ‘provider’ position

EHO/CBHO leadership reject the position laid out in the NHP which depicts the Government as the main actor and sole purchaser of health services for Myanmar. Leadership fear that EHO/CBHOs will be denied the ability to make their own decisions on what health services should be provided to the ethnic people and would be at risk of being controlled by the Government. Participants often stated that the Ethnic Health System must remain independent until a political peace agreement has been reached. Moreover, the provider role does not decentralise decision making in health planning, and does not give the ethnic people an opportunity to build their own capacity as one respondent hopes for:

*‘For the National Health Plan, we don’t want the Government to influence the Ethnic Health System... give a chance to ethnics to do it themselves... empower the Ethnic Health System.’ (IDI7, Central Office Coordinator).*

Disagreement with being depicted as a health provider, leads to EHO/CBHOs refusing integration into the MoHS health system. The NHP does state that purchasing of health services will be made by ‘a small semi-autonomous body steered by a board on which key stakeholders, such as MoHS, General Practitioner’s Society, EHOs and Civil Society, will be represented’ [11]. However, this is yet to occur in some ethnic territories. Until there is a tangible decentralisation of decision making in health planning, the EHO/CBHO health system will continue to remain parallel to that of the MoHS.

EHO/CBHOs remain interested in coordinating and collaborating with the MoHS to improve health outcomes. This has been seen in the national programs such as for malaria control:

*‘We use the National Program, the guidelines, but we’ve got the funding from the NGOs, international NGOs and local NGOs …*. ’ *(IDI11, Leadership).*

Collaboration has been possible without integration into the MoHS under the NHP. Nevertheless, each EHO/CBHO will have a slightly different approach to collaboration with the MoHS as explained by a participant:

*‘Some people are doing convergence with different strategies. Some closely, some a little far … But we try to balance each other.’ (IDI11, Leadership).*

Ultimately, all EHO/CBHOs agree upon the Rocket Ship model of convergence; until there is peace in Myanmar, the health systems should coordinate and work together to reach mutual goals whilst maintaining independence.

## Discussion

**Fig. 1:**
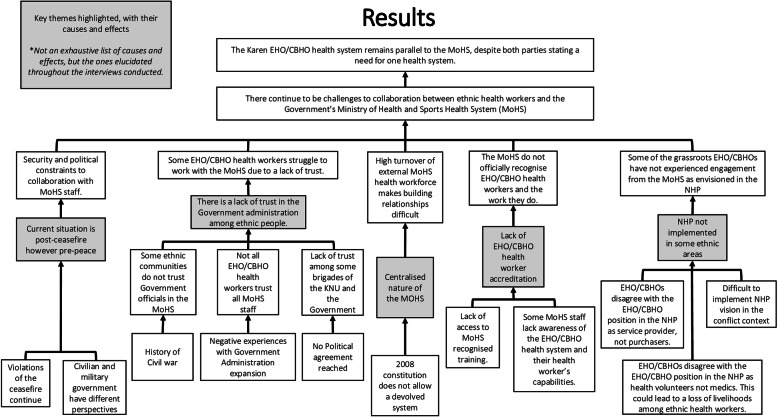
Results displayed in an analytical problem tree (source: author)

The key findings to the research have been illustrated in Fig. 1 below, an analytical problem tree to outline the challenges ethnic health workers have had in working with the MoHS.

### Implications

Some of the challenges to collaboration found in this study confirm findings discussed in a previous report in 2016, before the publication of the NHP by the MoHS: a lack of trust, the centralised nature of the MoHS and the lack of health worker accreditation [2]. The NHP outlined engagement with EHO/CBHOs in order to support one national health system. However, the core challenges to collaboration and convergence of health systems persist after over 3 years of NHP implementation. Although there have been improvements in the relationship between health workers in the Ethnic and Government health system, challenges expressed in the interviews evidence deeper roots, all of which are related to the lack of peace in Myanmar.

The health system cannot be removed from the political context in which it is situated. For health systems to have a role in state building, they must improve social cohesion through equitable access to health care, focusing on marginalised communities [27]. Some state the convergence agenda could have mutual goals in both improving health outcomes and peacebuilding, or ‘health as a bridge to peace’ [28]. However, participants stated that ‘for this country, it doesn’t work’ (IDI4, Leadership) as there primarily needs to be political will for peace. Bridges to peace can be built, whether peace can be attained is primarily based on political will.

Unfortunately, the MoHS and EHO/CBHOs are unable to materially affect the peace process. Instead EHO/CBHOs must follow the peace process and the interim political arrangements ‘in accordance with a federal democratic system’ [29]. If the EHO/CBHOs and the MoHS were to move ahead of the political process, with EHO/CBHOs integrated into a centralised MoHS, it would be difficult to return to a decentralised organisational structure should a politically decentralised Federal Union be created (IDI4, Leadership). Integration is not just about health system reform, it is a political reform process. EHO/CBHO integration with the MoHS takes away the legitimacy of EAOs and their governance structures, affecting the patron-client relationship between ethnic people and EAOs. EHO/CBHOs signing onto the NHP and becoming part of the MoHS may be perceived that the EHO/CBHO is then a part of the Myanmar Government. Given the lack of trust among ethnic people in the Government administration, this could have an impact on EHO/CBHOs access to ethnic communities. Hence the necessity for a gradual convergence of health systems that follows the peace process.

One key theme that emerged from the interviews is that all of Myanmar, including Kayin state, is not a post-conflict context. Health systems strengthening should therefore be conflict sensitive. Often health systems strengthening consolidates and supports the legitimacy of the Government through providing better health services [30]. Myanmar’s Government is not a neutral actor but one of the many stakeholders in Myanmar’s civil war. In these conflict contexts, ‘neither health system nor state are impartial bystanders’ [31]. Externally driven, donor led, strengthening only the Government’s health system is therefore legitimatising one stakeholder over another.

International donor engagement with the NHP has led to the gradual disengagement with EHO/CBHOs. This has become more prominent since the election of the civilian NLD Government. Additionally, for any international or national agency to work in Myanmar they must sign a Memorandum of Understanding (MoU) with the Government, and accept any changes made by the Government to their programs. Changes include the addition or removal of geographical locations which could be to distribute aid throughout the country, or due to political sensitivities in conflict-affected areas [32]. Therefore EHO/CBHOs in Kayin state have not signed an MoU with the Government nor the NHP, and consequently do not have direct access to international aid given to Myanmar for strengthening the health system and must receive funds through other organisations who have signed the MoU (IDI7, Central Office Coordinator).

Although there is an argument to support governmental health systems in development aid in order to facilitate early recovery and bridge the humanitarian-development gap [33], the conflict-sensitive approach to engagement would be to support all political actors and their governance structures [34]. Health systems strengthening should therefore ‘strengthen capacity at national and sub-national levels’ [30]. Both health systems have merits and therefore need to learn from each other in order to reach UHC (IDI5, Leadership). However, EHO/CBHO leadership felt that the NHP implies UHC can only be met by one health actor, that of the Government (IDI3, Leadership). In conflict contexts, the UN Office for the Coordination of Humanitarian Affairs (UNOCHA) suggest all actors should work together to attain collective outcomes as the ‘New Way of Working’. This allows for bridging of the humanitarian-development gap whilst also allowing organisations to maintain their independence from political actors [33]. Humanitarian and development organisations, as well as MoU signatories and non-signatories, can therefore collectively work together to achieve national goals without the obligation to integrate into the MoHS health system.

The importance of the EHO/CBHOs and how they have improved access to healthcare can’t be overemphasised. Of course, there are limitations to what the EHO/CBHO health system can provide in comparison to the MoHS health system. Participants described experiences of a lack of resources; reduced access to some medications and vaccines; reduced access to training hospitals; and reliance on external donor monetary support. Nevertheless as the MoHS health system struggles with a lack of human resources in ethnic areas [11], EHO/CBHO medics should be recognised as a valuable part of the health workforce. Most Karen EHO/CBHOs are composed of indigenous health workers based in the communities they serve. These indigenous medics can therefore be relied upon to support the ethnic communities because ‘they are from there, they born there, they grew up there, they will be there always, until they die’ (IDI4, Leadership). Local EHO/CBHO health workers also understand local languages and culture helping improve healthcare access for ethnic Karen people.

### Limitations

Only health workers in the EHO/CBHO health system were interviewed. In order to understand the challenges to collaboration, both the EHO/CBHO and MoHS perspective is needed. MoHS staff were not contacted due to concerns of participant security. The MoHS perspective is not only crucial for holistically understanding the challenges to collaboration, but participants stated that MoHS inclusion could have improved understanding between the two health systems (IDI6, IDI8, Central Office Coordinators). Triangulation with documents written by the MoHS, such as the NHP, was used to gain some perspective of the MoHS. Similarly, perspectives from donors were out of the scope of this study and not obtained due to time constraints. Future research into the MoHS and donor perspective is vital for further understanding of these complex health systems.

The next limitation of this study is the lack of inclusion of Kayin state field health workers in other EHO/CBHO organisations, such as the Burma Medical Association (BMA) or the KDHW. The choice to focus on BPHWT was due to their focus on sustainable primary healthcare, and that they are not officially affiliated to a political group. Leadership from each EHO/CBHO interviewed have a slightly different perspective on how closely EHO/CBHOs should work together. It would have been beneficial to explore how this influences field operations. However, all EHO/CBHO leadership discuss the same common themes regarding the core challenges to working with the MoHS health system.

Additionally, interviews with health workers from all townships would have been insightful with regards to the local situation in each region. However, time constraints and health worker availability did not allow this. Instead, experiences from BPHWT health workers in both KNU controlled and mixed governance territories was obtained and allowed insight into the different kinds of situations.

Finally, this research cannot be automatically generalised to other contexts worldwide or within Myanmar. This is because each state in Myanmar has a different political context, even each region within Kayin state will vary. Nevertheless, the results provide insight to the situation in Kayin state, where certain similarities can be drawn among other EHO/CBHO providers.

## Conclusions

Interviews with health workers in the EHO/CBHO health system revealed the challenges they face in working with the MoHS health system. All five themes that emerged from the transcripts have underlying roots in the lack of peace in Myanmar. Further research into the MoHS perspective would provide further clarity. Additionally, further research focusing on the impact of Covid-19 is crucial to reassess the challenges to collaboration between ethnic and governmental health systems.

EHO/CBHO health worker accreditation and systemic commitment to coordination between EHO/CBHOs and the MoHS will ease the challenges experienced in peer-to-peer and program level collaboration. Until there is a genuine peace accord, which is acceptable to all actors, the EHO/CBHO and MoHS health systems will inevitably remain parallel. EHO/CBHOs want a Federal Union where the health system is devolved, equitable and accessible to all ethnic people.

The study suggests that health systems cannot be ‘integrated’ or ‘converged’ without peace, nor can the health sectors meaningfully influence the peace process.

Pragmatic external actors engaging with health in Myanmar should understand this context. Often the aid sector applies a one-size-fits-all approach to seemingly ‘post-conflict’ reconstruction. Until the conflict ceases, aid given to one stakeholder over another is not neutral and aid agencies should consider this imbalance and review the application and direction of aid.

## Figures and Tables

**Fig. 2:**
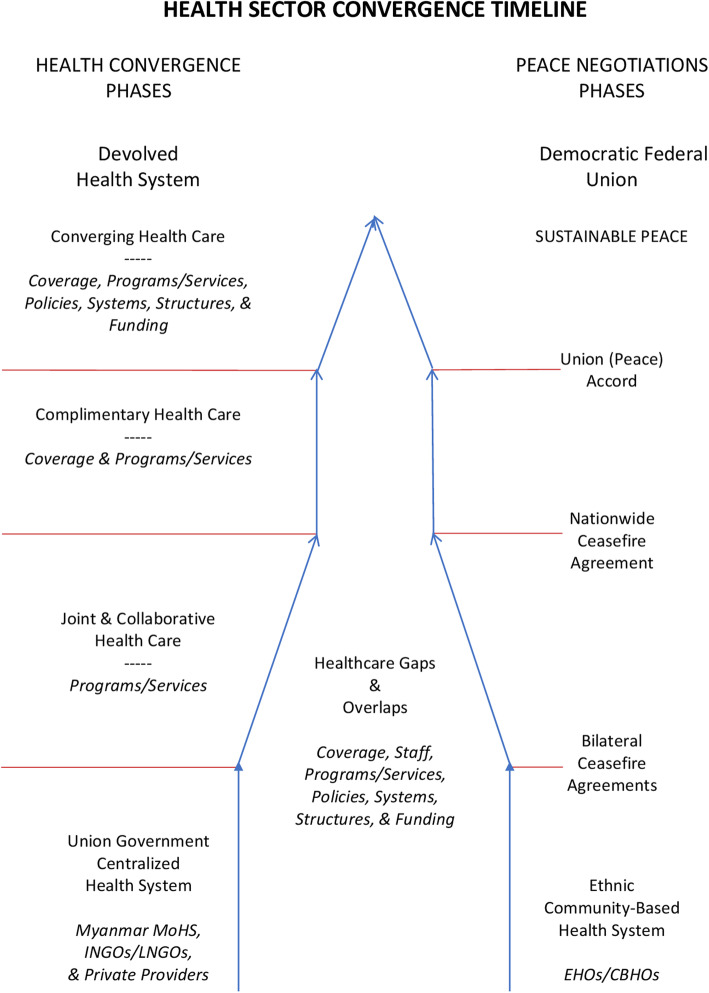
HCCG ‘Rocket-ship Model’

## Data Availability

The datasets generated and analysed during the current study are not publicly available due to the transcripts containing sensitive material and any identifiable factors that may compromise the identity of participants. Anonymised matrixes of coded quotes are available from the corresponding author on reasonable request.

## Abbreviations

ANC: Antenatal Care
BMA: Burma Medical Association
BPHWT: Back Pack Health Worker Team
CBHO: Community-Based Health Organisation
EAO: Ethnic Armed Organisation
EHO: Ethnic Health Organisation
EPHS: Essential Package of Health Services
EPI: Expanded Program for Immunisations
HCCG: Health Convergence Core Group
INGO: International Non-Governmental Organisation
KDHW: Kawthoolei (Karen) Department of Health and Welfare
KNU: Karen National Union
MoHS: (Myanmar) Ministry of Health and Sports
MoU: Memorandum of Understanding
NCA: Nationwide Ceasefire Agreement (2015)
NGO: Non-Governmental Organisation
NHP: National Health Plan
NLD: National League for Democracy
RHC: Rural Health Centre
UHC: Universal Health Coverage
UNOCHA: United Nations Office for the Coordination of Humanitarian Affairs
VHC: Village Health Committee
WHO: World Health Organisation
